# The role of forensic and legal systems in suicide prevention: Implications for Israeli healthcare policy

**DOI:** 10.1186/s13584-026-00773-0

**Published:** 2026-07-29

**Authors:** Gadi Lubin, Liat Keysar Gersht, Ligat Shalev

**Affiliations:** 1 The Jerusalem Mental Health Center, 96 Harav Raphael Katsenelbogen Street, Jerusalem, Israel; 2 The Medico-Legal Compass, 13 Rabi Akiva Street, Herzliya, Israel; 3 Hebrew University Faculty of Medicine, Jerusalem, 91120 Israel

**Keywords:** Law, Policy, Suicidality, Psychiatry, Malpractice, Court ruling, Forensic

## Abstract

**Background:**

Suicide is a leading cause of death among young people worldwide and remains a significant cause of mortality across all age groups, especially in older adults. The underlying causes of suicide span various disciplines and are deeply influenced by societal attitudes, which fluctuate between glorification and severe condemnation of suicide. This study aims to evaluate the role of court rulings on suicidal behavior, with particular attention to implications for healthcare policy in Israel.

**Methods:**

We searched the national court rulings databases for alleged psychiatry malpractice in suicide-related cases and of pre-trial settlements from 1985 to 2023. For court decisions, we collected patients’ socio-demographic and clinical characteristics, lawsuit characteristics, and the quality of medical documentation and decision-making processes. For pre-trial settlements, we collected data about lawsuit characteristics. Two researchers reviewed the qualitative data, while the quantitative data was analyzed with descriptive statistics.

**Results:**

Over the 38-year period, there were 20 psychiatry malpractice court lawsuits related to patient suicides, including 17 deaths and 3 life threatening attempts. Of these, 12 (60%) of cases were won by the plaintiff, and eight (40%) by the defendant. In 14 cases, the suicides occurred in inpatient units and in six cases in community settings. The number of lawsuits notably increased over time for suicide events, with 17 pre-court settlements between 1985 and 1997, and 56 cases in each of the subsequent periods (1998–2010 and 2011–2023).

**Conclusions:**

The number of malpractice lawsuits related to suicide in Israel is increasing over time. Our findings, which show a rise in both litigation and pre-trial settlements, may put pressure on psychiatrists to avoid certain kinds of work, especially in the public sector. Such a shift could have broader effects on the quality of suicide prevention efforts and overall mental health care. The study highlights the structural tension between retrospective legal accountability and prospective clinical and public-health approaches to suicide prevention. Addressing this challenge requires balanced policy responses, including strengthening patient-safety and risk-management systems, improving clinical documentation and decision transparency, fostering constructive collaboration between legal and mental health systems, and considering alternative compensation mechanisms that reduce adversarial pressures while supporting affected families.

## Background

Suicide is a leading cause of mortality across the age spectrum [1, 2]. Exploring the complex causes of suicide events covers a broad spectrum of disciplines, including philosophical, sociological, psychological, psychiatric, and general medical dimensions [3]. A suicidal event is a tragic fatal act of a person experiencing unbearable mental pain [4]. This event, in many cases, is an expression of a mental disorder [5]. Yet, suicidality is also influenced by the attitude of society and its institutions towards the phenomenon. The spectrum of attitudes in different societies, spans between one pole that demonstrates glorification and considers suicide a noble act such, as in India, to the opposite pole that criticizes it extremely [6, 7]. The effect of these positions on suicide rates - high and low, respectively, have been demonstrated throughout history [8, 9].

Several factors may improve the approach to preventing suicide. Health policy can have a significant effect on medical care across all fields, particularly in psychiatry [10]. A unique example in psychiatry is the contribution of forensic involvement in designing a “zero suicides during hospitalization” vision. This policy accelerated the creation of “sterile” spaces, as much as possible, free of lethal means. For example, this includes preventing self-hanging in toilets and showers by removing objects that could be used to hang oneself, such as wall hooks [11]. Another relevant factor is the quality of documentation of the therapeutic efforts. In the “accreditation language”, the documentation process in psychiatry in practice defines a “stop and check” time, that provides an important thinking and planning period [12].

According to Durfkheim (1990), altruistic suicide occurs in circumstances of “over-sense of belonging” of the individual in a society [13]. A person is willing to sacrifice himself for group goals. In many cases, the reference group to identify with and to sacrifice himself for is the family. Suicide might be based on a motivational background to solve a humiliating economic hardship, led by a sense of vulnerability, helplessness, loss of control and fatal narcissistic injury [14].

A version of altruistic suicides may be reflected by the effect of life insurance compensation on suicide rates. In every country, there is a fixed “refractory” period, which begins in the signing date of the policy, and is defined as one in which prior to its end there is no coverage for a suicide event. The duration of this period differs between countries, and usually ranges from one to three years. Several works demonstrated up to 50% increase in suicides in the month immediately following the end of this period, compared to the months preceding it [15].

### The theoretical perspective that guides the study: Regulation through litigation

There is an ongoing debate regarding the role of litigation in shaping public policy [16]. This debate, spanning both scholars and public health advocates, can help frame the issue, place it on the health policy agenda, and address the tension between regulation and the provision of adequate care, thereby encouraging self-regulation [17]. However, rather than functioning as a purely corrective regulatory mechanism, increasing volumes of litigation may generate self-reinforcing dynamics, whereby heightened legal pressure shapes clinical practice in ways that may introduce new risks and, ultimately, lead to additional claims. One manifestation of this process is defensive medicine [18]. This perspective is particularly relevant to the present study, which examines malpractice claims related to suicide, and may help explain evolving trends in psychiatric practice over time, with implications for both current and future clinical decision-making.

In the current study, we analyze 39 years of malpractice claims related to suicide in Israel, including both court rulings and out-of-court settlements. Our findings have important implications for health policy, offering an empirical perspective on regulation through litigation in the context of suicide-related malpractice claims.

## Methods

This study used a mixed-methods design, mixing quantitative and qualitative approaches. The study was conducted in two stages. In the first stage, we searched for all court rulings on claims of alleged malpractice in psychiatry inpatient and outpatient suicide-related cases conducted between the years 1985 to 2023. Earlier cases were excluded due to incomplete or insufficient documentation. We also did not include cases after 2024, as the most recent years include cases that remain in open procedural status. The database search words used included: suicide, suicidality, hospitalization, psychiatry, damages, and medical malpractice. We limited the search until 2023, because some more recent cases may not have settled yet, which could distort our findings. The database used for this search was Nevo, a comprehensive legal resource that provides access to court decisions from all levels of the Israeli judiciary.

It is important to mention that in Israel, trials do not involve a jury, but rather a single professional judge or a panel of professional judges, depending on the type of trial [19]. This may impact on the legal proceedings somewhat, and should be kept in mind. In most countries, a jury is involved with malpractice cases, which adds an element of unpredictability to the proceedings.

The following data was collected from the charges and court decisions (approval or rejection):

1. Sociodemographic characteristics of the suicidal patient: age, gender, and employment status prior to the event.
2. Clinical data: psychiatric diagnoses (psychotic spectrum/ personality disorders/ anxiety and/or depression disorders), suicide attempt outcome (fatal/ non-fatal but life-threatening), and location of the event (inpatient/ community).
3. Claim characteristics: claim outcome (plaintiff prevailed/ defendant prevailed), lawsuit classification (tort law/ preliminary criminal proceedings), credentials of the defendant (psychiatrist/ psychologist/ institute), compensation awarded, and documented consideration that led to court decision (free text).
4. Quality of medical documentation: motion expressions related to the clarity and detail of medical records (free text).

The second stage of the research focused on similar cases that had not gone to trial, but rather were settled out of court. These data spanned the period 1985–2023, and were collected from the database of the Internal Fund of the Israeli Governmental insurance, managed by Inbal Insurance Company LTD. This database covers approximately 90% of psychiatric departments nationwide. Data collection included cases characteristics, as well as the time interval from the suicidal event to the filing of the lawsuit and to the final settlement.

The qualitative materials- including the documented considerations underlying court decisions and statements regarding the clarity and detail of medical records, were reviewed to facilitate comprehensive learning and in-depth understanding of the cases. After that, they were rated and analyzed by the selected codes using explanatory content analysis [20]. To achieve interrater reliability, two researchers analyzed the material separately to ensure the trustworthiness of the results. In case of disagreement, further discussions were held until agreement was reached. Descriptive statistics were used to describe the quantitative data.

## Results

The study’s results are presented in two sections. The first examines court rulings on alleged malpractice claims in psychiatry-related suicide cases from 1985 to 2023. The second analyzes trends in forensic involvement and pre-court settlements from the equivalent years.

### Court rulings (1985 to 2023)

**Fig. 1:**
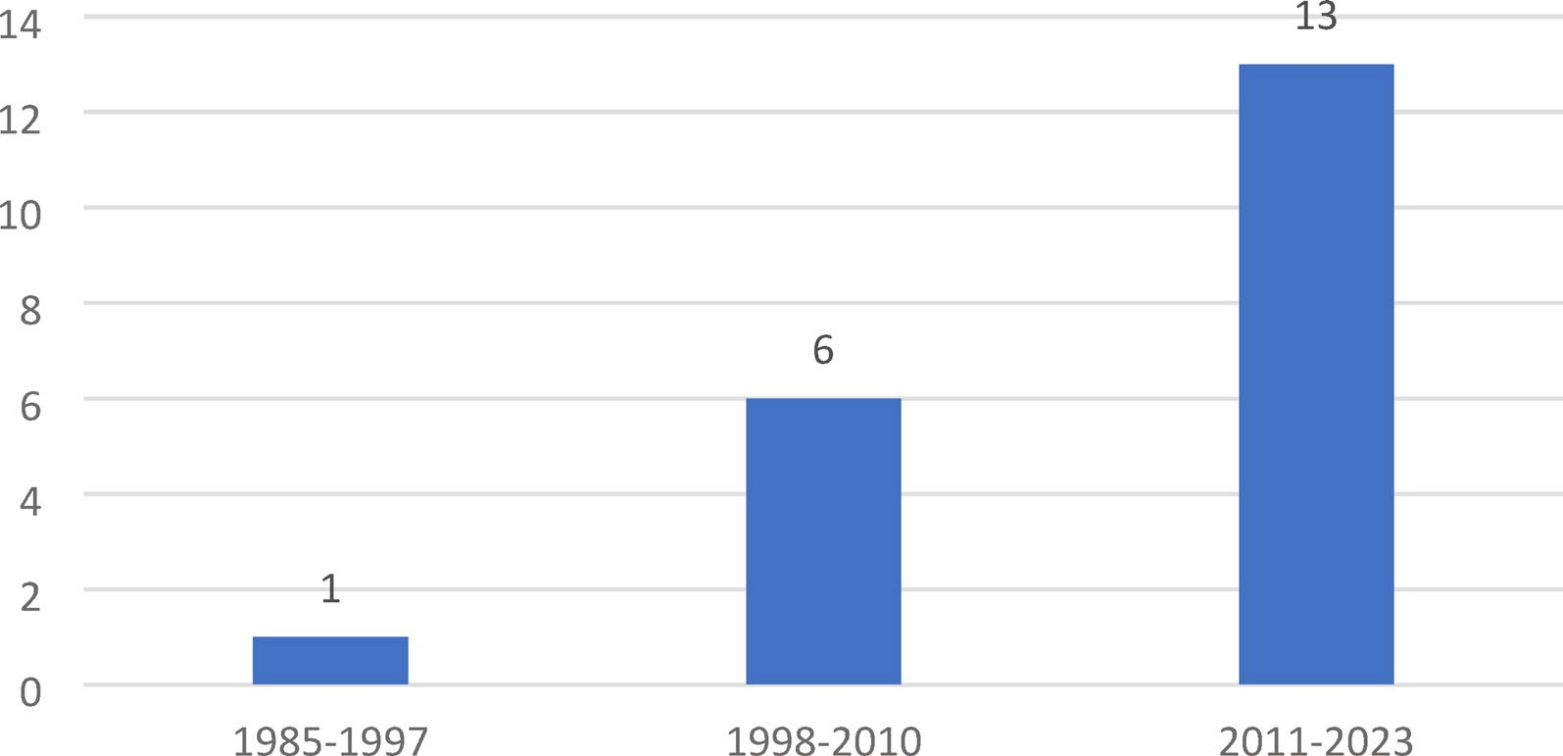
Distribution (no.) of psychiatry malpractice court rulings related to patient suicides by year, 1985–2023 (*n* = 20)

Over the 38-year period there were 20 psychiatry malpractice court rulings related to patient suicides. Among them, one took place between 1985 and 1997, six between 1998 and 2010, and the majority- 13 cases, took place between 2011 and 2023, reflecting a significant multi-year elevation in the rate of judgements in that field (Fig. 1).

Patient cases included 10 women and 10 men who ranged in age from 15 to 62 years old at the time of suicide attempt. Among 13 cases for which employment status information was available, eight (61%) of patients were unemployed.

Every patient had a psychiatric diagnosis: 13 (65%) were identified within the psychotic spectrum, two (10%) had personality disorders, and five (25%) were diagnosed with anxiety and/or depression. In 17 (85%) of the cases, the suicide attempts were fatal, and three (15%) were life-threatening but did not result in death. 14 (70%) events occurred on inpatient units and six (30%) in community settings.

Among the 20 cases, the plaintiff prevailed in 12 (60%), while the defendant prevailed in 8 (40%). All proceedings analyzed fell within tort law (i.e., the relief sought was monetary compensation). Eight of the court decisions were appealed. Of these, seven ultimately resulted in compensation being awarded, either through settlement or by affirming the lower court’s ruling. Of the 20 cases, 18 (90%) were directly targeted against psychiatrists, and two (10%) against psychologists. For the approved cases, the compensation ranged between 15,500 and 619,000 USD (adjusted from NIS to 2025 USD). Eighteen cases (90%) where a particular clinician was named also targeted an institution (mainly hospitals).

**Fig. 2:**
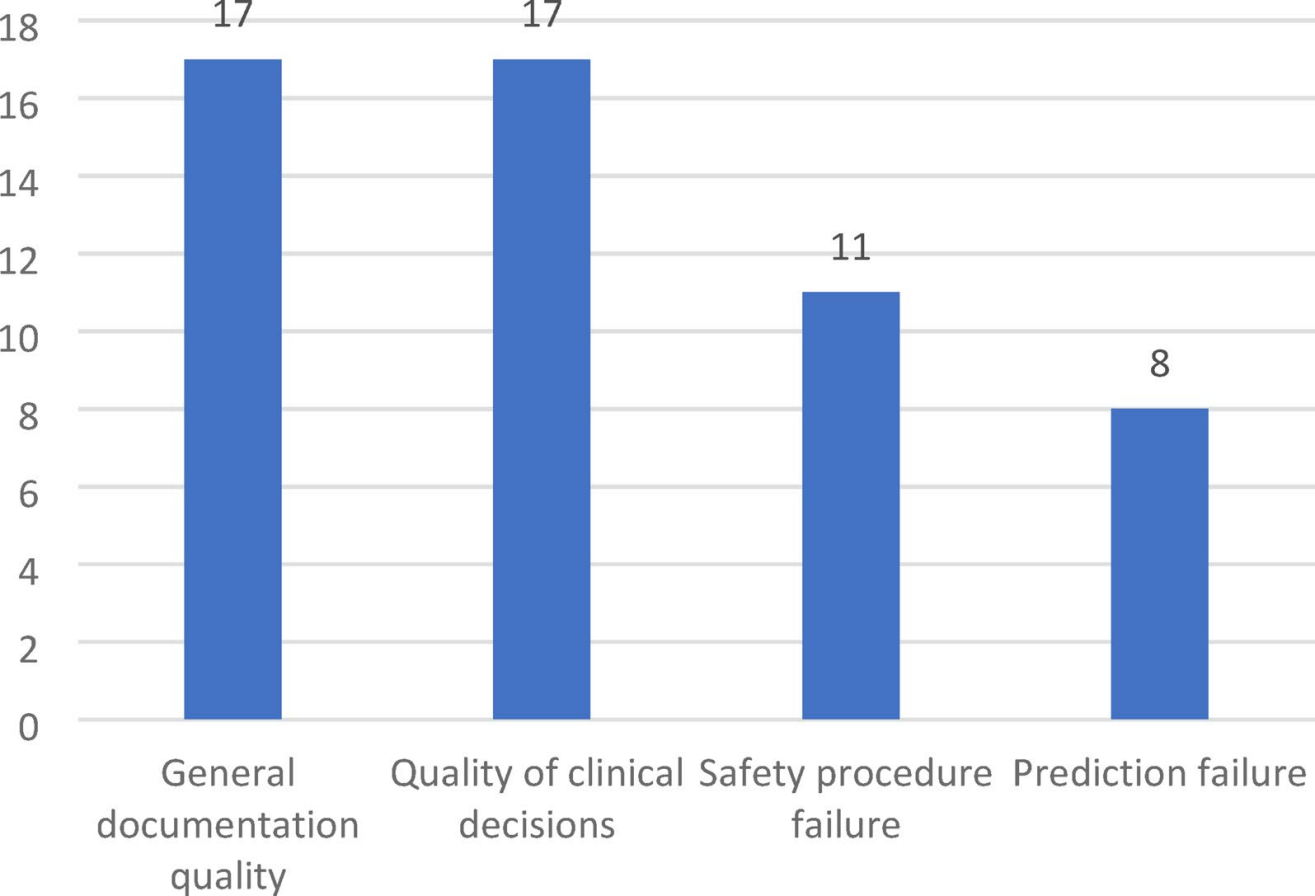
Distribution (no.) of courts’ main claims related to the quality of the medical documentation, 1985–2023 (*n* = 12; More than one category is possible per case, and more than one count is possible per category)

For the 12 cases won by the plaintiff, we found four main categories for the courts’ main claims related to the quality of the medical documentation (more than one category is possible per case, and more than one count is possible per category). The first category included 17 general notes regarding the medical records’ documentation quality. Among them, nine (53%) criticized the quality of the documentation in a general sense, five (29%) mentioned issues with the sufficiency of the history-taking, and three (17%) relied on retrospective history-taking, which generally reflects insufficient real time documentation. The quality of clinical decisions was another dominant group of court considerations, with a total of 17 mentions. Six of these mentions (35%) were general statements, and 11 (65%) referred to an inappropriate move from a locked/secured department to one with a more open policy, or to permitting parole (visits home followed by a return to the hospital), omission of necessary restrictions, omission of necessary forced admission, or mistaken releases from a psychiatric emergency room. A third group of statements (10 notes) related to a lack of implementation of safety procedure. Finally, the last category included seven statements about a failure to predict the suicide (Fig. 2).

**Fig. 3:**
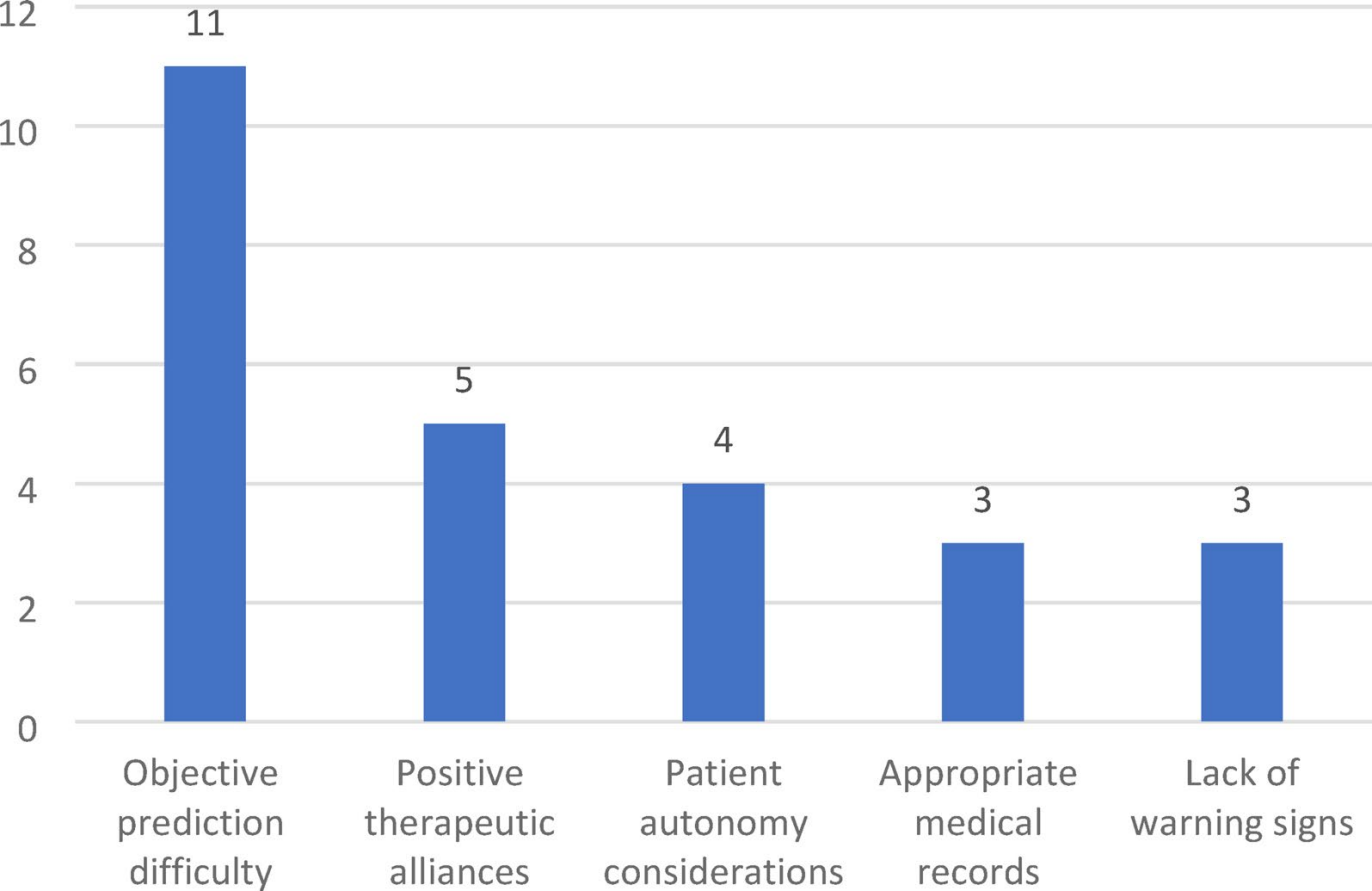
Distribution (no.) of courts’ court’s main considerations in cases decided in favor of the defendant, 1985–2023 (*n* = 8; More than one category is possible per case, and more than one count is possible per category)

Of the 20 cases that went to trial, eight were won by the defendant. The court’s main considerations in those cases included (more than one category is possible per case, and more than one count is possible per category): 11 general statements about the challenges of making reasonable and objective predictions; five statements that the evidence showed a positive therapeutic alliance; four mentions that patient autonomy was respected; three mentions of an appropriate medical record; and three mentions regarding the lack of clear “warning signs” (Fig. 3).

### Pre-trial settlements (1985 to 2023)

**Fig. 4:**
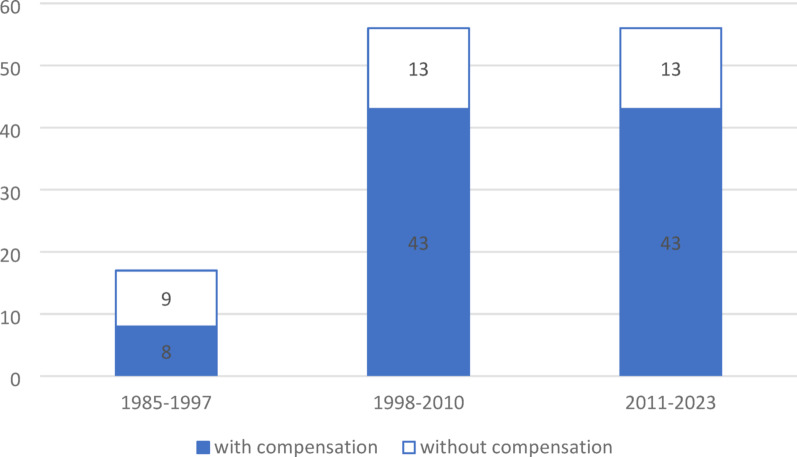
Distribution (no.) of pre-trial settlements related to suicide events by year categorized by cases with and without compensation, 1985–2023 (*n* = 129)

**Fig. 5:**
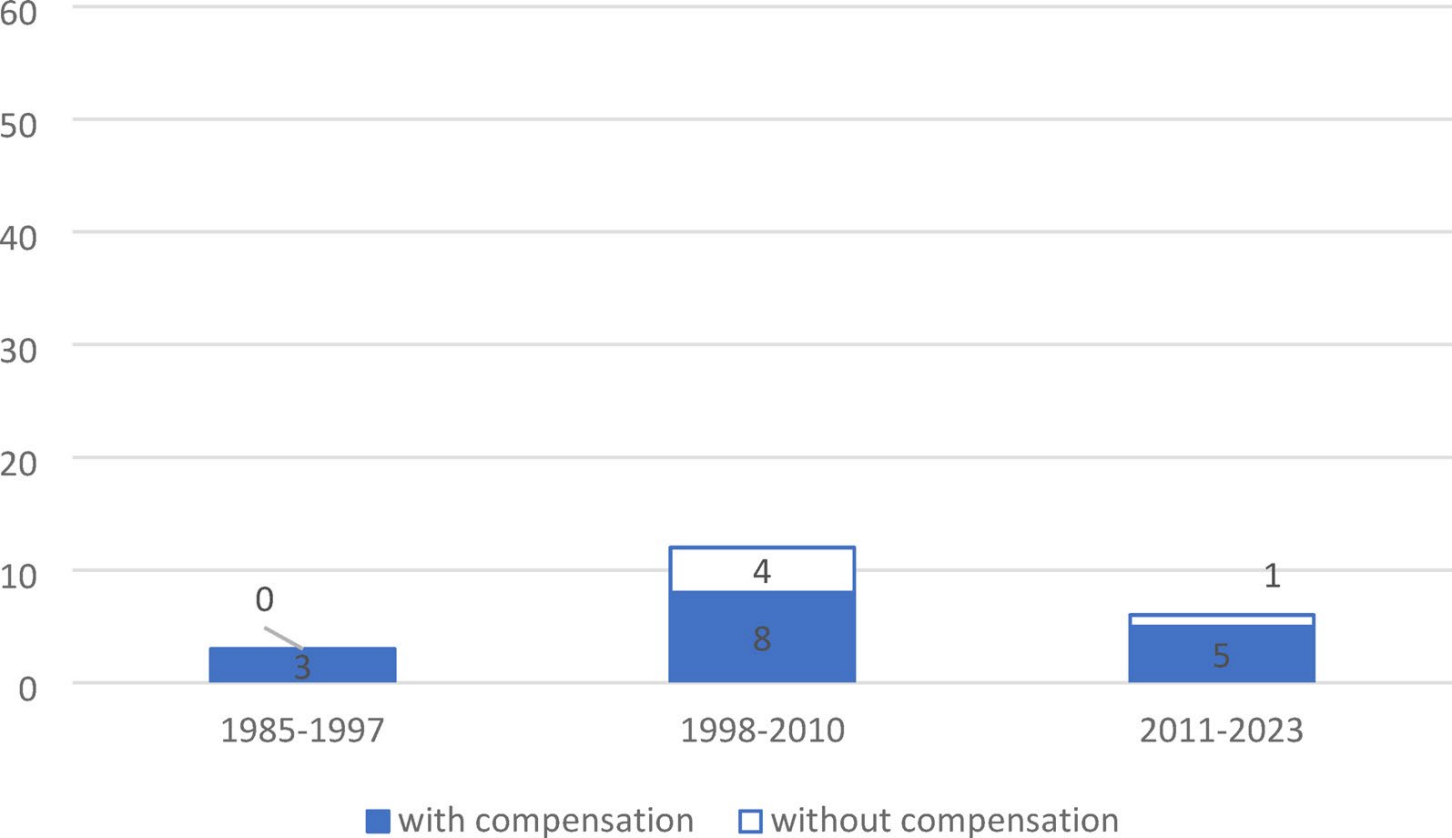
Distribution (no.) of pre-trial settlements related to suicide attempts by year categorized by cases with and without compensation, 1985–2023 (*n* = 21)

We also examined pre-trial settlements related to suicide events and suicide attempts between 1985 and 2023. There was a clear increase in the number of pre-trial settlements, with or without compensations, over time for both suicide events and suicide attempts. While we included only complete cases, we note that for suicide events, 23 cases between 2011 and 2023 remain open procedures, whereas for suicide attempts, the status of 4 cases remained open procedures. The time lapse between the suicidal event and the lawsuit ranged from several months to 7 years (Figs. 4 and 5).

## Discussion

We found and analyzed all malpractice court rulings regarding suicide attempts and completed suicides during the years 1985 to 2023 in Israel, as well as the pre-trial settlements from the same period. This analysis is informed by the theoretical perspective of regulation through litigation, which conceptualizes legal processes as a mechanism that shapes clinical behavior and health policy beyond formal regulatory frameworks [16]. We found 20 relevant court cases, of which 12 (60%) were won by the plaintiff, and eight (40%) by the defendant. The number of lawsuits related to suicide events increased over time, with 17 pre-court settlements recorded between 1985 and 1997, compared with 56 cases in each of the subsequent periods (1998–2010 and 2011–2023). Our findings reflect a clear trend toward more litigation over the years. Of the approved legal cases, the court’s main issues with the documentation related to the quality of the chart in general, and in particular the documentation of the medical history-taking. Additionally, the quality of clinical decision-making was criticized, such as inappropriate move from a secured department or allowing a patient home visits during the hospital stay. Of the cases won by the defendant, the most common court findings were that the documentation had been adequate and that the patient’s autonomy had been respected.

Our results show a clear trend toward more suicide-related lawsuits over time, both those that went to trial and those that were settled out of court. This finding contrasts with some other studies that examined similar issues in forensic systems of several other countries. For example, Martin-Fumadó and colleagues (2015) evaluated claims for alleged malpractice in psychiatry in Spain between 1986 and 2009. They found 94 claims, none of which involved suicide cases; 78% of these were processed through the courts [21]. Another study reviewed the published literature on malpractice lawsuits and medical board discipline from 1990 to 2009. They found that psychiatrists face an increased risk of disciplinary procedures by medical boards compared with other medical practices, and a lower risk of facing malpractice suits, whether decided in-court or settled out-of-court [22]. In our study, the increasing number of cases over time may be related to landmark laws passed in Israel, among them “Basic Law: Human Dignity and Liberty (1992)”, and “Patient’s Rights Law (1996)”. This highlights a fundamental tension inherent in litigation [16]. Specifically, it raises the issue of directionality: whether the increasing number of claims and court settlements related to suicide are driving changes in clinical practice, or whether the legal environment itself is shaping the rising trends observed.

The possibility of accountability-based audits and lawsuits has had significant beneficial effects on medical care in all fields, among them in Psychiatry [23]. We suggest that compared to previous eras, current times are characterized by a higher level of forensic involvement and impact on fundamental issues concerning the safety and the quality of patient treatment. Confidentiality of medical records, patient autonomy, the need for ethical approval and informed consent in research studies, and improved medical documentation standards are all vivid examples changes in law and policy that have benefited patients. However, the increased involvement of courts in psychiatry could also introduce meaningful obstacles to the practice.

Our findings show that among cases that went to trial, the majority were decided in favor of the plaintiff. On the face of it, this would seem to diminish the responsibility of the individuals as actors in their own right, and place more responsibility on the clinicians and the institutions treating them. This could contribute to a social and cultural climate in which the suicidal person’s image is of a victim and not of an event generator, promoting suicide stories that are easy to identify with [24]. This identification with the narrative of being a victim might spread among vulnerable individuals through identification with the act itself, to the point of imitating it. Therefore, we suggest that careful preservation of personal responsibility to one’s suicidal behavior and avoiding forensically projecting it on to others, is optimal and balanced both ethically and forensically and may eventually reduce suicidality.

A legal approach that externalizes responsibility to others has additional undesired effects, such as preferences of therapists for “safe” treatments or “safe” patients. An example is demonstrated in the study by McPherson and colleagues (2021). In their research, they examined the attitudes of the public and the medical staff in Canada regarding medical assistance in dying. Although they found a public preference for medical assistance in dying, they found that health professionals prefer hospice and palliative care. They concluded that these differences stem from the growing concern among professionals about the legal and regulatory implications of assistance in dying [25]. Other studies have shown that psychiatrists who have had patients who commit suicide have changed their professional practice in light of this experience [26, 27]. This finding may reflect insufficient consideration of hindsight bias- the tendency to view events as having been predictable after they have occurred, which can amplify psychiatrists’ sense of failure and guilt and may lead to the attribution of unjustified legal responsibility [28]. Moreover, private sector therapists are independent in their patient selection and can choose to accept only “safe” patients. Together with disparities in financial compensation, this creates a significant motivation to leave already threatened public services [26, 27].

Unemployment and financial crises are widely recognized as significant risk factors for suicide, impacting individuals without psychiatric backgrounds [29, 30], and even more so those with psychiatric histories [31, 32]. This study showed both high rates of unemployment and significant compensation payment. While our data do not include information on patients’ personal motives or intent, we offer a cautious conceptual reflection: it is theoretically possible that the visibility of cases involving significant compensation may contribute, in some circumstances, to what Durkheim termed *altruistic suicide*- that is, self-harm motivated by a perceived desire to benefit one’s family financially. This interpretation should be understood strictly as a hypothesis rather than an empirical conclusion. Future research examining individual-level motivations and perceptions would be required to evaluate this possibility.

Notwithstanding, it is of great importance to emphasize the need to provide multidimensional support for all families who lose their loved ones in suicidal incidents, as well as economic support if needed [33]. This can be achieved by implementing the “no fault” approach, known in some Scandinavian countries, in which various medical treatment events, which ended tragically, entitle the relevant patients with compensation, without legal proceedings and tort law [34]. In the Israeli context, such a model could potentially build upon existing risk-management mechanisms within the Ministry of Health and the institutional role of Inbal Insurance, which already oversees liability coverage for public healthcare institutions. A structured compensation fund, administered through a centralized governmental or quasi-governmental framework, could provide standardized, modest financial support alongside access to specialized therapeutic services for bereaved families. Importantly, decoupling compensation from adversarial legal proceedings may also enhance transparency and facilitate organizational learning, as clinical events could be examined within a quality-improvement framework rather than through prolonged litigation. Such reforms would require careful regulatory design but could reduce legal pressures while strengthening suicide prevention and patient-safety strategies.

In the current study, the court most frequently criticized the security policies and related decisions. This contract may be better understood as a distinction between retrospective legal reasoning and prospective public-health reasoning. Legal analysis is inherently retrospective: it examines events after they have occurred and focuses on liability, foreseeability, and accountability. By contrast, clinical and public-health approaches are prospective in nature, emphasizing prevention, population-level risk management, system design, and decision-making under conditions of uncertainty. These differing temporal and conceptual orientations may generate divergent expectations regarding acceptable risk and appropriate intervention. The forensic approach demonstrated in our findings tended to reflect a relatively linear hierarchy of safety interventions, ranging from ambulatory care, considered least secure, to progressively more restrictive open and locked inpatient settings. In contrast, clinical decision-making weighs a broader and more nuanced set of considerations, including the patient’s preferences, autonomy, therapeutic alliance, self-image, and the potential impact of restrictive environments on recovery. This difference creates a clear structural tension: within retrospective legal reasoning, more restrictive environments may appear preferable, particularly with the benefit of hindsight [35, 36], whereas from a prospective clinical perspective, overly restrictive measures may undermine long-term recovery and engagement. Defensive medicine in this context may therefore exact a meaningful toll on therapeutic processes. A tangible example is a suicide that occurred in a hospital shortly after a patient’s request for a home visit, motivated by an “intense sense of longing for his wife and children”, was denied due to being deemed “not ready.” We will never know whether granting the visit might have altered the outcome (the quotes are from the medical record). At the same time, suicide events occurring during home visits are also documented [37]. These examples illustrate the inherent uncertainty in prospective clinical decision-making, which differs fundamentally from retrospective legal evaluation.

Documenting a broader set of considerations may help in legal proceedings. The extensive ruling notes concerning the quality of relevant medical records, both as general impressions and in detailed specific aspects is an indication regarding the forensic significant contribution to this crucial aspect [38]. Like many other studies, ours emphasizes yet again the importance of adequate documentation- especially to capture the complexity of medical decision-making as it was seen at the time of the decision.

A suicide event shakes the fundamental beliefs in the lives of those who are exposed to it. Consequently, this characteristic leads to a common denominator of all those involved in the field, to do everything they can to prevent it. Examining suicide events from a clinical perspective is different than examining them from a forensic perspective. In the former, the emphasis is on improving care going forward; in the latter, the emphasis is on assigning blame and accountability. Aside from the important contribution of forensic thinking and various rulings to the quality and safety of treatment, gaps have arisen over the years in relation to clinical thinking, which carry a significant price tag. It requires from both sides, openness, and significant adjustments. This study has addressed some of these issues. One practical step could be the establishment of forensic-psychiatric consultation panels aimed at malpractice prevention and risk management, creating a structured space for dialogue before cases escalate to litigation. In addition, strengthening structured collaboration between legal and mental health systems through joint training initiatives or formalized communication channels may help align clinical and legal perspectives, reduce adversarial dynamics, and promote shared learning. This study highlights the importance of such integrative approaches.

This study has several limitations. First, the inclusion criteria were limited to malpractice claims, which constitute much less than 1% of total national suicide cases over the same period. The chosen methodology is grounded in the significant influence that court decisions and statements exert on various legal processes, including compensation settlements, pre-court agreements, and the guidelines that inform psychiatric staff consultations regarding security policies. These elements also shape the public and media narratives concerning suicide prevention. To minimize this limitation, we supplemented our data by also including pre-trial settlement agreements. The current analysis includes 20 cases from 1985 onward. Earlier cases were not included due to incomplete or insufficient documentation. Thus, the sample represents all accurately documented and legally accessible cases identified during the examined period. Given the limited number of relevant cases, we advise future research to expand this methodology into an international context. Such an approach would allow for cross-cultural comparisons and enhance the generalizability of the findings. We also recommend evaluating this topic by examining the perceptions of various stakeholders, including policymakers, suicide researchers, legal experts, psychiatrists from both public and private sectors, and families and patients. Finally, we believe that examining an integrated medico-legal-public health framework would represent an important contribution. Such work could help clarify how legal accountability mechanisms influence clinical risk-management decisions and shape system-level suicide prevention strategies.

## Conclusions

This paper examines the role of policy in suicide prevention through the lens of lawsuits and court rulings related to psychiatric malpractice in suicide cases. Grounded in the theoretical perspective of regulation through litigation, it conceptualizes legal processes as mechanisms that shape clinical behavior and influence health policy. We reviewed all relevant rulings in Israel between 1985 and 2023, identifying 20 psychiatry malpractice lawsuits connected to patient suicides. Our findings indicate a significant increase in such cases starting in 1986, along with a rise in pre-trial settlement payments from 2009 onward. These trends suggest a growing involvement of the legal system in suicide-related claims. The findings may help explain why psychiatrists are increasingly leaving the public sector and how such legal pressures could potentially impact suicide rates. Additionally, the results highlight the need for policy reforms, including interventions like more secure hospitalization settings, to prevent suicides. This study contributes to expanding the understanding of suicide prevention by exploring the intersection of legislation, court rulings, and mental health policy, offering insights into how policy changes could help mitigate this phenomenon.

## Data Availability

The datasets used and/or analyzed during the current study are available from the corresponding author upon reasonable request.
